# The Effect of Magnetoelastic Anisotropy on the Magnetization Processes in Rapidly Quenched Amorphous Nanowires

**DOI:** 10.3390/ma17051141

**Published:** 2024-02-29

**Authors:** Cristian Rotarescu, Sorin Corodeanu, Costică Hlenschi, George Stoian, Horia Chiriac, Nicoleta Lupu, Tibor-Adrian Óvári

**Affiliations:** National Institute of Research and Development for Technical Physics, 47 Mangeron Boulevard, 700050 Iași, Romania; scorodeanu@phys-iasi.ro (S.C.); gstoian@phys-iasi.ro (G.S.); hchiriac@phys-iasi.ro (H.C.); nicole@phys-iasi.ro (N.L.)

**Keywords:** magnetic anisotropy, cylindrical amorphous nanowires, switching field, micromagnetic modelling

## Abstract

In this paper, we report for the first time on the theoretical and experimental investigation of Fe_77.5_Si_7.5_B_15_ amorphous glass-coated nanowires by analyzing samples with the same diameters in both cases. The hysteresis curves, the dependence of the switching field values on nanowire dimensions, and the effect of the magnetoelastic anisotropy on the magnetization processes were analyzed and interpreted to explain the magnetization reversal in highly magnetostrictive amorphous nanowires prepared in cylindrical shape by rapid quenching from the melt. All the measured samples were found to be magnetically bistable, being characterized by rectangular hysteresis loops. The most important feature of the study is the inclusion of the magnetoelastic anisotropy term that originates in the specific production process of these amorphous nanowires. The results show that the switching field decreases when the nanowire diameter increases and this effect is due to the reduction in anisotropy and in the intrinsic mechanical stresses. Moreover, the obtained results reveal the importance of factors such as geometry and magnetoelastic anisotropy for the experimental design of cylindrical amorphous nanowires for multiple applications in miniaturized devices, like micro and nanosensors.

## 1. Introduction

Cylindrical amorphous nanowires are rapidly solidified amorphous magnetic wires with nanosized diameters and with a large aspect ratio (length/diameter) [1]. Nanowires with cylindrical symmetry are expected to play an important role as the building blocks of three-dimensional (3D) information technologies such as shift registers, magnetic recording, spintronics, logic gates, and sensing architectures [2,3,4]. The inclusion of physical sensors is particularly important for future 3D Internet of Things nanoscale platforms [5,6,7]. Magnetic domain walls, which separate two regions of opposite magnetization in a nanowire, can be manipulated and used to encode information for storage [8,9] or to perform logic operations [10]. This manipulation entails the controlled nucleation, depinning, and propagation of domain walls along 3D vertical and horizontal tracks.

Thin films and flat thin strips were the first choices for studying the domain walls, due to the simplicity of fabricating them by nanopatterning and of simulating their behavior with commonly available micromagnetic codes.

However, the attention has partly shifted to nanowires with circular cross-section, namely, nanocylinders [11], because their geometry emphasizes a number of favorable properties concerning the domain walls motion in comparison with thin and flat magnetic strips.

In this context, computational micromagnetics has become an indispensable tool for the theoretical investigation of magnetic nanostructures [12], accelerating the translation of basic research into microelectronic technologies [13]. Modelling and design of glass-coated cylindrical amorphous nanowires is a key research area needed to support their technological applications.

The motivation of this work is to understand the magnetic behavior and properties of ultrathin cylindrical amorphous nanowires by investigating, both theoretically and experimentally, samples with the same diameters. Previous simulations have been performed on idealized samples that did not match the actual diameters of the samples on which the experimental studies were performed. Therefore, the interpretation of the results was rather qualitative. Here, we aimed to simulate micromagnetically nanowires with the same diameters as the ones that we measured, this being the main novelty of this article.

In the present investigation, we have focused on the influence of magnetic anisotropy on the magnetization reversal process within magnetostrictive amorphous nanowires having various diameters, in the range of 100 to 200 nm. We performed micromagnetic simulations of the magnetic hysteresis loops of individual amorphous nanowires in order to reveal their magnetic properties arising from the contribution of geometrical effects (diameter) and to interpret the experimental results. Magnetoelastic anisotropy gives rise to important effects in rapidly solidified amorphous nanowires due to the coupling between magnetostriction and internal mechanical stresses inherent in their preparation process. The magnetization reversal process is expected to proceed by the nucleation and propagation of a magnetic domain wall, as has been previously reported experimentally for rapidly solidified amorphous nanowires [14].

These ultrathin cylindrical amorphous wires exhibit many advantages in comparison with other types of nanowires prepared by electrodeposition or by various lithography techniques: much longer wire length, reduced cost, larger mechanical stresses that result in significant magnetoelastic contributions [15], more suitable shape for fast and ultrafast domain wall propagation (vs. planar samples), they are obtained as single nanowires (vs. electrodeposited arrays of nanowires [16]), and the presence of the amorphous phase (i.e., the absence of magnetocrystalline anisotropy). The last advantage allows us to make a rigorous study of the role played by the magnetoelastic anisotropy in their magnetic behavior.

Here, a micromagnetic study of the axial hysteresis loops in cylindrical glass-coated amorphous nanowires is expected to provide a comprehensive description of the magnetization reversal process in an individual nanowire, giving important information concerning the switching field and the dimensional effects on their overall magnetic behavior.

In the next sections, we will present the methodology used in this research (Section 2), while in Section 3 we will discuss and interpret the obtained results. Finally, we present the conclusions and the future potential of our research in this topic.

## 2. Materials and Methods

**Figure 1:**
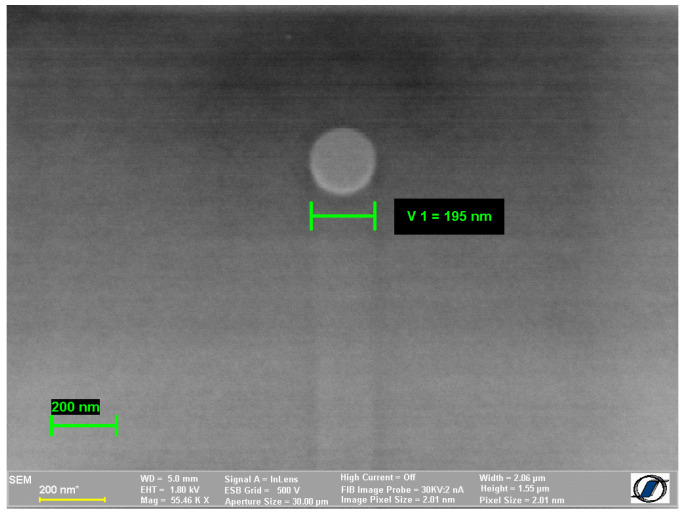
High-magnification SEM image of a rapidly solidified Fe_77.5_Si_7.5_B_15_ amorphous nanowire being 195 nm in diameter.

The investigated samples are cylindrical amorphous nanowires with the composition Fe_77.5_Si_7.5_B_15_ (alloy with large and positive magnetostriction λ = 25 × 10^−6^). This type of glass-coated amorphous nanowire has been prepared by an improved variant of the glass-coated melt spinning method at the National Institute of Research and Development for Technical Physics Iași [14,15]. In this method, when the alloy pieces are melted, the closed end of the glass tube becomes soft, which allows a glass capillary to be drawn. The molten metal stream flows through the continuously drawn and spooled glass capillary, the result being a metal–glass ensemble which is cooled with a cooling fluid below the inductor. This process allows the production of very long wires (of the order of km in length), similar to the case of glass-coated microwires. Moreover, the very small transverse dimensions establish the right conditions for the rapid quenching of the metallic alloy, the final result being the amorphous glass-coated nanowire. In the last years, some improvements were made to the method, which refer to the drawing speed of the wires, to the suppression of the mechanical vibrations in order to avoid breaking the continuity of such thin wires, and to the tailoring of the specific process parameters needed to prepare them [17]. The magnetostriction has been measured using the small-angle magnetization rotation (SAMR) method that has been previously used for thin cylindrical magnetic wires [18] and has been found to be 25 × 10^−6^. Figure 1 illustrates a high-magnification scanning electron microscopy (SEM) image of such a sample. Here, we aimed to present a larger image (10-times larger magnification) in comparison with the SEM image from reference [19], only to show how small these wires are. In reference [19], the actual magnetic wire is just the bright dot in the middle. All the rest is glass coating. Images have been acquired using a CrossBeam system NEON 40 EsB FIB-SEM microscope from Carl Zeiss (Oberkochen, Germany) with thermal Schottky field emission, equipped with EDS, in-lens, SE, and EsB detectors. The electron beam resolution was 1.1 ÷ 2.5 nm for U = 20 ÷ 1 kV. For acquiring the SEM images, we used the in-lens and EsB detectors under the following conditions: electrons accelerating voltages (EHT) of 1.8 kV and a working distance (WD) of 5 mm.

The axial hysteresis loops of the as-prepared amorphous nanowires have been measured by means of an inductive technique specifically designed for these ultrathin cylindrical amorphous wires with a magnetically bistable behavior [19], i.e., in which the axial magnetization is reversed in just one step when the axial magnetic field reaches a threshold value called switching field, H*. The method employs a digital integration technique that allows one to measure noise-free loops [20]. The hysteresis loops were measured by applying a magnetic field parallel to the easy axis of the nanowire. The field was applied from H_max_ value to −H_max_ value with a step field in order to obtain the descending branch of the hysteresis loop and, after, from −H_max_ value to H_max_ for the ascending branch of the hysteresis.

The components of the measuring system are: the magnetizing solenoid, the system of pickup coils, a low-noise amplifier, a function generator, and a data acquisition board [19]. The magnetizing solenoid is powered by a function generator through a high-power bipolar amplifier and generates magnetic fields of up to 30 kA/m. The pickup coils are connected in series opposition in order to avoid any induced voltage in the absence of the sample and have the following characteristics: 1 cm in length and 1570 turns, wound with enameled 0.07 mm copper wire on a ceramic tube. The voltages induced in the pickup coil are proportional to the applied magnetic field and are amplified using a low-noise preamplifier in order to obtain a measurable value and a high signal-to-noise ratio (SNR). The amplified induced voltages are digitized using a four-channel simultaneous data acquisition board. The acquired signals are processed using LabVIEW-based software (version 2010) and the sampling frequency is between 800 kHz and 10 MHz (with 5000 to 62,500 points/loop at 160 Hz).

For the theoretical approach, we performed a simpler model able to provide details of the magnetization switching and to quickly perform a large number of simulations. The micromagnetic analysis of the highly magnetostrictive Fe_77.5_Si_7.5_B_15_ amorphous nanowires was developed with the aim of interpreting the experimental hysteresis loop data obtained by measurements on samples with similar diameters. Although the magnetic behavior of these ultrathin samples is expected to be similar to that of the well-known glass-coated amorphous microwires with larger diameters [21], here, we aimed to analyze the peculiarities of amorphous nanowires by means of both micromagnetic modelling and hysteresis loop measurements.

The implementation of the different geometric and magnetic characteristics is conducted with the finite-difference package MuMax3 [22], with low memory requirements, opening up the possibility of very large-scale micromagnetic simulations in limited time and using inexpensive hardware. This software solves the time- and space-dependent magnetization evolution of the nanowires and allows a smooth numerical approximation of the glass-coated nanowires with cylindrical shapes.

The developed model takes into account the dimensions (diameter and length) and the magnetic properties (the saturation magnetization M_S_ and the exchange constant A) of these nanowires and allows one to study the dependence of the magnetization reversal process on their dimensions and, at the same time, to determine their remanence and coercivity. In the simulations, we have considered the values for highly magnetostrictive Fe_77.5_Si_7.5_B_15_ amorphous nanowires: µ_0_ M_S_ = 1.6 T and A = 1.5 × 10^−11^ J/m [23]. Certainly, these two parameters, saturation polarization and exchange constant, are very important for the micromagnetic model and were obtained by VSM measurements for the amorphous nanowires with the composition Fe_77.5_Si_7.5_B_15_. In all the simulations, the field H was applied parallel to the easy axis of the nanowire.

We have chosen to simulate such amorphous cylindrical samples with the length of 2 µm, which ensures a sufficiently large aspect ratio, and the diameters in the same range as the samples we have employed for the hysteresis loop measurements in order to have a complete, theoretical and experimental, characterization of the nanowires with this composition. The finite-discretization method (FDM) used in the simulations assumes that the magnetic region is subdivided into a regular cuboid mesh where the cell discretization size (equal to 2 nm) is smaller than the exchange length $l_{ex}=\sqrt{A/K_{eff}}$. Here, the effective anisotropy K_eff_ includes the contribution of the shape anisotropy introduced by the demagnetization field, as well as the magnetoelastic anisotropy K_me_. The results were obtained for the damping parameter α = 0.5 since we are modelling a rectangular hysteresis loop in a quasi-static approximation. We have determined the equilibrium configuration of the magnetization for every applied field using this large value of the damping, which is only available to treat the static cases (the hysteresis loops simulations). It is very important to mention that if one wants to study the domain wall propagation and to determine the domain wall velocity, then one needs to set very low values of the damping (from 0.01 to 0.05) [24]—this range describes the dynamic case.

**Figure 2:**
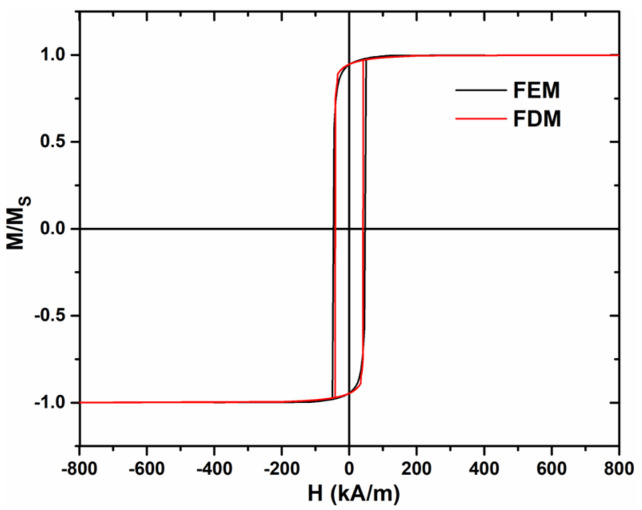
Micromagnetic simulations of the hysteresis loop using both methods (FEM and FDM).

The FDM is a very popular numerical tool with two main advantages: it gives the solution of micromagnetic equations and allows the application of very fast algorithms (e.g., fast Fourier transform (FFT) accelerated demagnetization field computation). To have an idea about how the choice of the discretization method (FEM or FDM) affects the simulation results, we compared the two methods while keeping all the other parameters unchanged: length—1 μm, diameter—100 nm, and cell size—2 nm. Figure 2 shows the results of this comparison. One observes that the discretization method does not affect the simulated hysteresis loops.

The essential condition in the static micromagnetics approximation is a stable magnetization configuration realized by a minimum of a total magnetic energy (E_tot_) of the system with respect to its magnetization. In this way, we have determined the equilibrium configurations of the magnetization $\vec{M}$ for all the values of the applied magnetic field $\vec{H}$, solving the Landau–Lifshitz–Gilbert (LLG) equation [25]:

(1) $$\frac{\partial \vec{M}}{\partial t}=-\left|\gamma \right|\vec{M}\times {\vec{H}}_{\mathrm{eff}}+\left(\alpha /M_{S}\right)\vec{M}\times \frac{\partial \vec{M}}{\partial t}$$

where ${\vec{H}}_{\mathrm{eff}}=\frac{-1}{\mu_{0}}\frac{{\delta E}_{\mathrm{tot}}}{\delta \vec{m}}$ is the effective magnetic field.

The LLG Equation (1) determines the time variation of the magnetization ($\frac{\partial \vec{M}}{\partial t}$) considering the gyromagnetic ratio (*γ*), the Gilbert damping (*α*), the total magnetic energy (E_tot_), and the magnetization ($\vec{m}$) designed to a discretization element.

The effective magnetic field ${\vec{H}}_{eff}$ definition in the proposed model reflects the main interactions within the nanosized magnetostrictive amorphous cylinder:

E_tot_ = E_ex_ + E_dip_ + E_Zeeman_ + E_me_ (2)

where E_ex_ is the exchange energy, E_dip_ is the energy for dipolar interaction (magnetostatic nature), and E_Zeeman_ is the Zeeman energy, whilst E_me_ is the magnetoelastic contribution due to the magnetostrictive nature of the alloy (the last term contains the magnetoelastic anisotropy constant K_me_).

We have studied the evolution of the magnetic properties with the changes in sample diameter as well as the effect of the magnetoelastic term using both experimental and simulation approaches. The novelty of this study arises mainly from the unique opportunity to directly compare experimental hysteresis loop data with simulated ones, calculated for nanowires with the same diameters. In addition, the role of the magnetic anisotropies is highlighted using the capabilities of MuMax3, which allows one to simulate samples with a high aspect ratio. It is important to emphasize that the shape of the hysteresis loops (experimental or simulated) depends on the sample dimensions and composition.

## 3. Results and Discussion

The glass-coated amorphous nanowires with positive magnetostriction (Fe_77.5_Si_7.5_B_15_) prepared by melt spinning were studied theoretically by micromagnetic simulations and experimentally by hysteresis loop measurements and SEM in order to systematically analyze their magnetic behavior. Generally, we have several factors which influence the magnetic behavior in amorphous nanowires obtained by melt spinning: the direction of the applied field with respect to the easy axis, the anisotropy (magnetostatic and magnetoelastic contributions), and the dimensions of the nanowires (length and diameter).

As mentioned above, the investigated samples show a one-step magnetization reversal along the wire axis when the axial magnetic field reaches the value of the switching field. This is an important characteristic, named magnetic bistability, and its experimental proof is given by the presence of a rectangular hysteresis loop.

**Figure 3:**
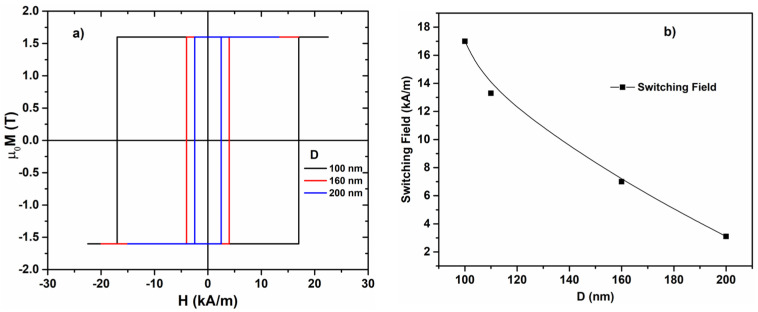
(**a**) Experimental hysteresis loops of Fe_77.5_Si_7.5_B_15_ amorphous nanowire and (**b**) switching field dependence on the nanowires with different diameters.

Figure 3a,b show the experimental axial hysteresis loops of the cylindrical glass-coated nanowires with the diameters of the metallic nucleus (D) of 100, 160, and 200 nm and the switching field dependence on the metallic nanowire diameter extracted from these experimentally determined hysteresis loops.

The magnitude of the switching field and its rapid decrease with the increase in the metallic nanowire diameter agree with a magnetoelastic origin of the magnetic anisotropy in these novel ultrathin magnetic wires. As the diameter increases, the level of internal stresses induced during the wire preparation by rapid solidification decreases, leading to an overall smaller value of the magnetoelastic anisotropy and switching field.

**Figure 4:**
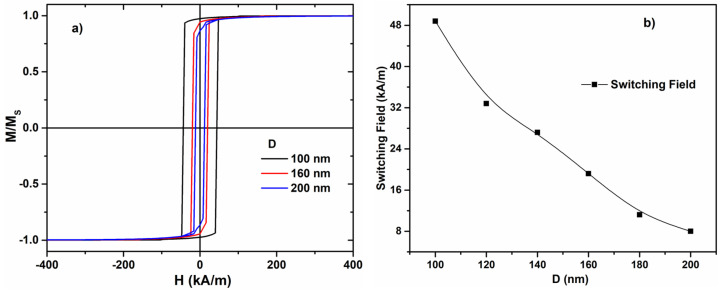
Micromagnetic simulations of: (**a**) hysteresis loops of Fe_77.5_Si_7.5_B_15_ amorphous nanowires (the diameters D are 100, 160, and 200 nm) and (**b**) switching field dependence on the diameter.

For the same composition, we performed micromagnetic simulations using the finite-differences method in order to calculate the hysteresis loops and to extract the variation of the switching field with the nanowire diameter (Figure 4a,b). The theoretical results clearly confirm that when the nanowire diameter D increases (100 nm → 200 nm), the value of the switching field decreases. This behavior is similar to the one observed experimentally for all the samples with the composition Fe_77.5_Si_7.5_B_15_.

The observed magnetic bistability and the magnitude of the switching field and its rapid decrease with the increase in the diameter of the metallic nanowire are in agreement with the magnetoelastic origin of the preponderant magnetic anisotropy. This is now proven both experimentally and theoretically.

**Figure 5:**
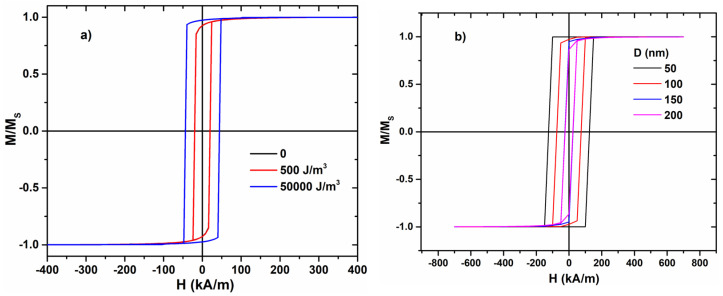
Simulated axial hysteresis loops for: (**a**) a wire with 100 nm in diameter (the effect of different values of K_me_) and (**b**) various diameters (D).

In order to have a reasonable correlation between the real conditions in which we measured the hysteresis loops and the simulation conditions, we considered a value of the internal mechanical stresses, i.e., those induced during the preparation of the nanowires, which correspond to a magnetoelastic anisotropy constant of 5 × 10^4^ J/m^3^ (see Figure 5a).

To substantiate the approach taken and the key role of the magnetoelastic anisotropy, we have calculated the effect of various magnitudes of the magnetoelastic anisotropy on the simulated loops. Figure 5a shows the simulated loops in the case of the nanowire with 100 nm diameter and 2 µm length for various levels of axial magnetoelastic anisotropy between 0 and 5 × 10^4^ J/m^3^. These limits include the entire range of potential values for the axial magnetoelastic anisotropy constant given by the relation $K_{me}=\frac{3}{2}\lambda \sigma$, the upper limit allowing axial tensile stress values of up to several GPa, which is the order of magnitude for the maximum stresses reported in this type of samples [26].

The effect of the magnetoelastic anisotropy has an important particularity, highlighted in Figure 5a: a low magnetoelastic anisotropy leads to a small value of the switching field when comparing magnetostrictive wires with the same diameter. This can be caused either by a smaller magnetostriction or by smaller internal stresses.

To consolidate the approximation taken, we included other simulations for various diameters but it is very important to mention that the effect is the same (the length is 2 µm and the magnetoelastic constant K = 50,000 J/m^3^): the switching field decreases when the diameter of the nanowire increases (Figure 5b).

One observes that magnetoelastic anisotropy constants up to 5 × 10^2^ J/m^3^ do not affect the value of the switching field, as shown in Figure 5a. However, the increment of K_me_ to 5 × 10^4^ J/m^3^ results in a drastic increase in the switching field, emphasizing its importance in the magnetization reversal process.

The calculated values of the switching field are larger than the experimental values measured for the samples with similar diameters, as shown in Figure 3a,b. This is a consequence of the conditions used in the simulations, where, initially, we have a uniform magnetization (saturated sample). Thus, magnetization reversal requires the nucleation of a new domain with reverse magnetization. The related magnetic domain wall will suffer depinning and will propagate at magnetization switching. The nucleation process entails larger fields which translate into higher values of the switching field. In the experimental case, the situation is completely different because there are already, at the ends of the nanowire, domains with reverse magnetization due to the demagnetization effect and these lead to smaller values of the required axially applied field, which drives the magnetization switching process (no nucleation is necessary).

Thus, the highly magnetostrictive amorphous nanowires have an important reduction in the magnetoelastic contribution (K_me_) with the increase in diameter, due to the fact that internal stresses induced during the preparation of the thicker nanowires are much smaller. This effect has been emphasized by both numerical simulations and hysteresis loop measurements for samples with the composition Fe_77.5_Si_7.5_B_15_, being supported by previous calculations of internal stresses in such materials and their variation with the nanowire diameter [26]. It is important to emphasize that this general trend of the magnetic behavior is determined solely by the magnetoelastic anisotropy, without the need to include the shape anisotropy in the analysis, which proves that the magnetoelastic contribution has the most important influence on the magnetic behavior of the rapidly solidified amorphous nanowires made from highly magnetostrictive alloys.

## 4. Conclusions

The micromagnetic analysis and experimental investigation conducted in this work are meant to reduce the gap between theoretical and experimental results, creating the right environment for the experimental design of cylindrical amorphous nanowires for multiple applications. Moreover, the results of this investigation improve the overall accuracy of the information on the magnetic behavior of this type of wires. The main novelty is the correlation between experimental results and simulation results in the case of amorphous nanowires with the same diameters and magnetic characteristics.

In addition, the roles played by the main anisotropies have been better pointed out by means of the capabilities of MuMax3, which can simulate samples with a high aspect ratio.

These results are important for accurately controlling and tailoring the magnetic behavior and properties of the nanometric amorphous wires prepared by rapid quenching from the melt. Thus, through a precise control of the nanowire diameters, one can adjust in a significative range the characteristics of their hysteresis loops, giving rise to the possibility of using these materials for technological applications, e.g., sensing elements in miniaturized sensors (micro and nano devices) or domain wall conduits for fast-moving domain walls in domain-wall-based logic devices with low power consumption.

Future work will focus on novel compositions, such as near-zero magnetostrictive ones, and novel structures, e.g., nanocrystalline, as well as on the effects of the glass coating reduction on the magnetic behavior of rapidly solidified nanowires.

## Data Availability

Data are contained within the article.
